# Favorable prognostic impact of *phosphatase and tensin homolog* alterations in wild-type isocitrate dehydrogenase and *telomerase reverse transcriptase* promoter glioblastoma

**DOI:** 10.1093/noajnl/vdad078

**Published:** 2023-06-28

**Authors:** Nayuta Higa, Toshiaki Akahane, Seiya Yokoyama, Ryutaro Makino, Hajime Yonezawa, Hiroyuki Uchida, Tomoko Takajo, Mari Kirishima, Taiji Hamada, Naoki Noguchi, Ryosuke Otsuji, Daisuke Kuga, Shohei Nagasaka, Hitoshi Yamahata, Junkoh Yamamoto, Koji Yoshimoto, Akihide Tanimoto, Ryosuke Hanaya

**Affiliations:** Department of Neurosurgery, Graduate School of Medical and Dental Sciences, Kagoshima University, Kagoshima, Japan; Department of Neurosurgery, Graduate School of Medical Sciences, Kyushu University, Fukuoka, Japan; Department of Pathology, Graduate School of Medical and Dental Sciences, Kagoshima University, Kagoshima, Japan; Center for Human Genome and Gene Analysis, Kagoshima University Hospital, Kagoshima, Japan; Department of Pathology, Graduate School of Medical and Dental Sciences, Kagoshima University, Kagoshima, Japan; Department of Neurosurgery, Graduate School of Medical and Dental Sciences, Kagoshima University, Kagoshima, Japan; Department of Neurosurgery, Graduate School of Medical and Dental Sciences, Kagoshima University, Kagoshima, Japan; Department of Neurosurgery, Graduate School of Medical and Dental Sciences, Kagoshima University, Kagoshima, Japan; Department of Neurosurgery, Graduate School of Medical and Dental Sciences, Kagoshima University, Kagoshima, Japan; Department of Pathology, Graduate School of Medical and Dental Sciences, Kagoshima University, Kagoshima, Japan; Department of Pathology, Graduate School of Medical and Dental Sciences, Kagoshima University, Kagoshima, Japan; Department of Neurosurgery, Graduate School of Medical Sciences, Kyushu University, Fukuoka, Japan; Department of Neurosurgery, Graduate School of Medical Sciences, Kyushu University, Fukuoka, Japan; Department of Neurosurgery, Graduate School of Medical Sciences, Kyushu University, Fukuoka, Japan; Department of Neurosurgery, University of Occupational and Environmental Health, Kitakyushu, Japan; Department of Neurosurgery, Graduate School of Medical and Dental Sciences, Kagoshima University, Kagoshima, Japan; Department of Neurosurgery, University of Occupational and Environmental Health, Kitakyushu, Japan; Department of Neurosurgery, Graduate School of Medical Sciences, Kyushu University, Fukuoka, Japan; Department of Pathology, Graduate School of Medical and Dental Sciences, Kagoshima University, Kagoshima, Japan; Center for Human Genome and Gene Analysis, Kagoshima University Hospital, Kagoshima, Japan; Department of Neurosurgery, Graduate School of Medical and Dental Sciences, Kagoshima University, Kagoshima, Japan

**Keywords:** PTEN, prognostic markers, TERTp, TERTp-wild-type glioblastoma

## Abstract

**Background:**

*Telomerase reverse transcriptase* promoter (*TERTp*) mutations are a biological marker of glioblastoma; however, the prognostic significance of *TERTp* mutational status is controversial. We evaluated this impact by retrospectively analyzing the outcomes of patients with isocitrate dehydrogenase (*IDH)-* and *TERTp-wild*-type glioblastomas.

**Methods:**

Using custom next-generation sequencing, we analyzed 208 glioblastoma samples harboring wild-type *IDH*.

**Results:**

*TERTp* mutations were detected in 143 samples (68.8%). The remaining 65 (31.2%) were *TERTp*-wild-type. Among the *TERTp*-wild-type glioblastoma samples, we observed a significant difference in median progression-free survival (18.6 and 11.4 months, respectively) and overall survival (not reached and 15.7 months, respectively) in patients with and without *phosphatase and tensin homolog (PTEN)* loss and/or mutation. Patients with *TERTp*-wild-type glioblastomas with *PTEN* loss and/or mutation were younger and had higher Karnofsky Performance Status scores than those without *PTEN* loss and/or mutation. We divided the patients with *TERTp*-wild-type into 3 clusters using unsupervised hierarchical clustering: Good (*PTEN* and *TP53* alterations; lack of *CDKN2A/B* homozygous deletion and *platelet-derived growth factor receptor alpha (PDGFRA)* alterations), intermediate (*PTEN* alterations, *CDKN2A/B* homozygous deletion, lack of *PDGFRA,* and *TP53* alterations), and poor (*PDGFRA* and *TP53* alterations, *CDKN2A/B* homozygous deletion, and lack of *PTEN* alterations) outcomes. Kaplan–Meier survival analysis indicated that these clusters significantly correlated with the overall survival of *TERTp*-wild-type glioblastoma patients.

**Conclusions:**

Here, we report that *PTEN* loss and/or mutation is the most useful marker for predicting favorable outcomes in patients with *IDH*- and *TERTp*-wild-type glioblastomas. The combination of 4 genes, *PTEN*, *TP53*, *CDKN2A/B*, and *PDGFRA*, is important for the molecular classification and individual prognosis of patients with *IDH*- and *TERTp*-wild-type glioblastomas.

**Key Points**

- Median OS varies in isocitrate dehydrogenase (*IDH*)- and *TERTp*-wild-type GBM with and without *PTEN* loss/mutation.
- *PTEN*, *TP53*, *CDKN2A/B*, and *PDGFRA* are important in the molecular classification of *IDH*- and *TERTp*-wild-type GBM.

**Importance of the study**

Biological markers for glioblastoma include *TERT* promoter (*TERTp*) mutations. As the frequency of *TERTp* mutations in glioblastoma is approximately 70%–90%, the relatively small number of *TERTp*-wild-type glioblastoma cases has limited the study of its molecular characteristics and prognostic factors. Here, we report that *PTEN* alterations are associated with favorable outcomes in patients with *TERTp*-wild-type glioblastomas. Using hierarchical molecular classification, we revealed 3 distinct clusters of *TERTp*-wild-type glioblastoma prognosis groups: good (alterations in *PTEN* and *TP53*, and lack of *CDKN2A/B* homozygous deletion and *PDGFRA* alterations), intermediate (alterations in *PTEN*, *CDKN2A/B* homozygous deletion, and lack of alterations in *PDGFRA* and *TP53*), and poor (alterations in *PDGFRA* and *TP53*, homozygous deletion of *CDKN2A/B*, and lack of alterations in *PTEN*). A combination of 4 genes (*PTEN*, *TP53*, *CDKN2A/B*, and *PDGFRA*) is important for the molecular classification and prognosis of patients with IDH- and TERTp-wild-type glioblastomas.

Glioblastoma (GBM) is the most common primary malignant brain tumor in adults and is classified as grade 4 by the World Health Organization (WHO). GBM’s relapse rate is very high, and median survival is typically only 10 to 11 months, even with multimodal treatment encompassing surgery, radiation, and chemotherapy.^1^ GBM is heterogeneous with a wide mutational spectrum.^2–4^ To obtain insights into the biology of this tumor and subsequently improve its diagnosis and treatment, the molecular classification of GBM has been intensifying.

*TERT* promoter (*TERTp*) mutations are collectively one of the biological and diagnostic markers for GBM.^5–7^ TERT is a reverse transcriptase catalytic subunit of telomerase that maintains telomere lengths.^8^ Telomere maintenance is essential for the unlimited proliferation of tumor cells and occurs in many cancer types via the reactivation of telomerase.^9–11^*TERTp* mutations lead to increased TERT expression and telomerase activation.^12^*TERTp* mutations are frequently observed in GBM. Recent studies indicated that 70%–90% of GBM genomes harbor *TERTp* mutations.^13–15^ In contrast, low frequencies of *TERTp* mutations were reported in Japanese groups.^16–20^ Thus, the frequencies of *TERTp*-wild-type GBM are higher in Japanese patients than in patients from other countries. Diplas et al. found that a subset of isocitrate dehydrogenase (*IDH*)- and *TERTp*-wild-type GBM utilized distinct genetic mechanisms of telomere maintenance driven by alternative lengthening in telomerase-positive cells displaying alterations in *ATRX* or *SMARCAL1* and *TERT* structural rearrangements.^21^ However, for *TERTp*-wild-type GBM, there is relatively less information, and the mechanism of telomere maintenance remains unknown. To the best of our knowledge, there have been only 3 publications dedicated to *IDH*- and *TERTp*-wild-type GBMs.^21–23^

In this study, we examined a cohort of *IDH*- and *TERTp*-wild-type GBMs with next-generation sequencing (NGS) using a custom gene panel that we recently reported.^16^ This study aimed to examine the clinical characteristics of patients with *IDH*- and *TERTp*-wild-type GBMs and provide a better understanding of the molecular profiles of *IDH*- and *TERTp*-wild-type GBMs.

## Materials and Methods

The current study adhered to the reporting recommendations for tumor marker prognostic studies (REMARK) guidelines. The completed checklist is provided in Supplementary Appendix 1.

### GBM Samples

Two hundred and eight formalin-fixed paraffin-embedded (FFPE) tumor tissue samples were collected from Kagoshima University, Kyushu University, and University of Occupational and Environmental Health. The study was approved by the Institutional Review Board of Kagoshima University (approval number: 180104) and complied with the tenets of the Declaration of Helsinki. Informed consent was obtained from all patients. Resected tumors were fixed with 10% phosphate-buffered formalin within 24 hours of sampling and routinely processed for paraffin embedding, followed by sections for hematoxylin and eosin staining. All tumors were originally classified according to the WHO 2021 classification. All tissues were histologically evaluated by board-certified pathologists (M.K. and A.T.) to ensure an estimated tumor cell content of ≥ 30%.

### Treatments

This was a retrospective study that included 208 patients with *IDH*-wild-type GBM with available molecular data between 2014 and 2022. We removed ≥90% of the tumor in 94 patients (45.2%) and < 90% of the tumor in 114 patients (54.8%). Additionally, 194 GBM patients were treated with temozolomide during radiotherapy, followed by temozolomide maintenance treatments. However, 14 patients were not treated because of poor clinical status attributed to factors such as advanced age or low Karnofsky Performance Status (KPS) scores.

### DNA Extraction and Quantification

For DNA preparation from FFPE samples, we used the Maxwell 16 FFPE Tissue LEV DNA Purification Kit (Promega, Madison, WI, USA, Cat#AS1130) according to the manufacturer’s instructions. Thereafter, DNA concentration was measured using a Qubit 3.0 Fluorometer dsDNA BR Assay Kit (Life Technologies, Grand Island, NY, USA, Cat#Q32850), and DNA quality was monitored using the QIAseq DNA QuantiMIZE Kit (QIAGEN, Reston, VA, USA, Cat#333414). The extracted DNA was diluted to 5–10 ng/μL as a template, and PCR was performed using the QIAseq DNA QuantiMIZE Kit (QIAGEN, Cat#333414).

### NGS

NGS was performed using the QIAseq Targeted DNA Custom Panel (QIAGEN, Cat#333525), as described previously.^16^ Amplicon sequences were aligned to the human reference genome GRCh37 (hg19) in the target region of the sequence. Data were analyzed using the QIAGEN Web Portal service (https://www.qiagen.com/).

### Data Analysis

We used OncoPrinter (cbioportal.org/oncoprinter), which is a tool in the cBioPortal for Cancer Genomics software system, to visualize and analyze our data.^24,25^ We analyzed the data using EZR (Easy R) (Saitama Medical Center, Jichi Medical University, Saitama, Japan), a graphical user interface of the R software (The R Foundation for Statistical Computing, Vienna, Austria). We compared the risk groups and patient characteristics using the chi-square (*χ*^2^) and Kaplan–Meier log-rank tests, respectively. We also performed univariate and multivariate Cox regression analyses. A *P*-value of <.05 was considered statistically significant. Additionally, unsupervised average linkage hierarchical clustering was applied to the NGS data obtained from the tumors based on Jaccard’s matching coefficient to calculate distances. This analysis was performed using the R open-source statistical computing language (v3.5.3), integrated development environment RStudio (v0.99.484), and the R packages nmf (v0.20.6), mass (v7.3–51.5), and stats (v3.2.2). Cluster analysis was performed using Euclidean distance and Ward.D2 linkage.

## Results

### Clinical and Genetic Factors Associated With *TERTp* Mutation Status

We identified 208 patients with *IDH*-wild-type GBM with available molecular data between 2014 and 2022. Within this cohort, *TERTp* mutations were detected in 143 tumors (68.8%). The remaining 65 (31.2%) were *TERTp*-wild-type. Clinical factors, including sex, average patient age, KPS score, extent of resection (EOR), and chemoradiotherapy, were not significantly different between *TERTp*-wild-type and *TERTp*-mutant GBMs (Table 1). Moreover, progression-free survival (PFS) and overall survival (OS) were not significantly different between *TERTp*-wild-type and *TERTp*-mutant GBMs (*P* = .481 and *P* = .449, respectively; Supplementary Figure 1A, 1B). Importantly, *PDGFRA* amplification and/or mutation and *TP53* loss and/or mutation were more common in patients with *TERTp*-wild-type GBMs than in those with *TERTp*-mutant GBMs (Table 1). Conversely, *epidermal growth factor receptor (EGFR)* amplification and/or mutation, and *PTEN* loss and/or mutation were more commonly observed in *TERTp*-mutant GBMs than in *TERTp*-wild-type GBMs (Table 1). There was no difference in the frequencies of *ATRX*, *SMARCA4*, and *ARID1A* mutations between patients with *TERTp*-*wild*-type and *TERTp*-mutant GBMs (Table 1).

**Table 1.** Background of Patients With and Without *TERTp* Mutation

Prognostic Factor		All (*n* = 208)	*TERTp* Mutation (*n* = 143)	*TERTp* Wild (*n* = 65)	*P*-value
Sex	male	123 (59.1%)	87 (60.8%)	36 (55.4%)	.543
	female	85 (40.9%)	56 (39.2%)	29 (44.6%)	
Age	70 years>	125 (60.1%)	91 (63.6%)	34 (52.3%)	.130
	70 years≤	83 (39.9%)	52 (36.4%)	31 (47.7%)	
KPS score	80 points≤	103 (49.5%)	75 (52.4%)	28 (43.1%)	.233
	80 points>	105 (50.5%)	68 (47.6%)	37 (56.9%)	
Resection	90 %≤	94 (45.2%)	67 (46.9%)	27 (41.5%)	.548
	90 %>	114 (54.8%)	76 (53.1%)	38 (58.5%)	
Chemoradiotherapy	Yes	194 (93.3%)	134 (93.7%)	60 (92.3%)	.768
	No	14 (6.7%)	9 (6.3%)	5 (7.7%)	
*CDKN2A/B* homdel		94 (45.2%)	66 (46.2%)	28 (43.1%)	.764
*NF1* loss and/or mut		49 (23.6%)	38 (26.6%)	11 (16.9%)	.159
*PTEN* loss and/or mut		134 (64.4%)	108 (75.5%)	26 (40.0%)	<.001^*^
*RB1* loss and/or mut		89 (42.8%)	61 (42.7%)	28 (43.1%)	1.000
*PDGFRA* amp and/or mut		43 (20.7%)	14 (9.8%)	29 (44.6%)	<.001^*^
*TP53* loss and/or mut		86 (41.3%)	49 (34.3%)	37 (56.9%)	.003^*^
*EGFR* amp and/or mut		69 (33.2%)	62 (43.4%)	7 (10.8%)	<.001^*^
*ATRX* loss and/or mut		48 (23.1%)	35 (24.5%)	13 (20.0%)	.595
*ARID1A* mut		6 (2.9%)	5 (3.5%)	1 (1.5%)	.668
*SMARCA4* mut		4 (1.9%)	2 (1.4%)	2 (3.1%)	.591

KPS, Karnofsky Performance Status; mut, mutation; amp, amplification; homdel, homozygous deletion.

^*^indicates statistical significance.

### Clinical and Genetic Factors Influencing Prognosis in *TERTp*-Wild-Type GBMs

In *TERTp*-wild-type GBMs, the most commonly altered genes were *TP53* (57%), *PDGFRA* (45%), *CDKN2A/B* (43%), *RB1* (43%), and *PTEN* (40%) (Supplementary Figure 2). First, we analyzed whether the identified genetic markers were prognostic markers in *TERTp*-wild-type GBM. Four clinical features, including sex, age, KPS score, and EOR, were reduced dimensionally via principal component analysis; one optimal feature set, named “clinical information” was subsequently created. Thereafter, we adjusted for covariates, including “clinical information,” in the multivariate Cox proportional hazards model. Notably, *CDKN2A/B* homozygous deletion and *PDGFRA* amplification and/or mutation were significant indicators of poor prognosis, as determined by our univariate analysis (hazard ratio [HR]: 2.16 [1.05–4.43], *P = *.036; and HR: 3.13 [1.48–6.63], *P = *.003, respectively; Table 2), but were not significant indicators of prognosis in our multivariate analyses. *PTEN* loss and/or mutation was a significant indicator of favorable prognosis, as determined by our univariate analysis (HR: 0.20 [0.08–0.46], *P < *.001; Table 2). In our multivariate analyses, *PTEN* loss and/or mutation was only an independent significant indicator of favorable prognosis in *TERTp*-wild-type GBM (HR: 0.25 [0.08–0.79], *P = *.018; Table 2). In contrast, *PDGFRA* amplification and/or mutation was a significant indicator of poor prognosis, as determined by our univariate (HR: 2.11 [1.03–4.31], *P = *.041) and multivariate (HR: 2.26 [1.04–4.91], *P = *.039) analyses in *TERTp*-mutant GBM (Supplementary Table 1).

**Table 2.** Genetic Prognostic Factors in *TERTp* Wild-Type GBM

	Univariate Analysis		Multivariate Analysis	
Prognostic marker	HR (95% CI)	*P*-value	HR (95% CI)	*P*-value
Clinical information	0.99 (0.98–1.01)	.298	0.99 (0.97–1.01)	.404
*CDKN2A/B* homdel	2.16 (1.05–4.43)	.036^*^	0.68 (0.25–1.90)	.464
*NF1* loss and/or mut	0.40 (0.12–1.31)	.130	0.70 (0.20–2.53)	.590
*RB1* loss and/or mut	0.83 (0.41–1.71)	.619	0.95 (0.42–2.14)	.907
*EGFR* amp and/or mut	0.19 (0.03–1.40)	.103	0.30 (0.04–2.48)	.266
*PDGFRA* amp and/or mut	3.13 (1.48–6.63)	.003^*^	1.65 (0.68–4.02)	.267
*TP53* loss and/or mut	1.55 (0.75–3.19)	.235	1.85 (0.83–4.10)	.130
*PTEN* loss and/or mut	0.20 (0.08–0.46)	<.001^*^	0.24 (0.08–0.77)	.016^*^

mut, mutation; homdel, homozygous deletion; amp, amplification.

^*^indicates statistical significance.

Second, we identified the clinical prognostic factors, which included analysis of the genetic markers for *PTEN* loss and/or mutation in *TERTp*-wild-type GBM. Our univariate analysis revealed that age (HR: 2.88 [1.38–6.00], *P = *.005), EOR (HR: 2.24 [1.06–4.73], *P = *.035), and *PTEN* loss and/or mutation (HR: 0.20 [0.08–0.46], *P < *.001) were significantly associated with prognosis (Table 3). Thereafter, we adjusted for covariates, including sex, age, KPS score, and EOR, in the multivariate Cox proportional hazards model. This analysis corroborated the finding that age and *PTEN* loss and/or mutation were independent prognostic markers of OS in patients with *TERTp*-wild-type GBM (HR: 2.69 [1.19–6.10], *P = *.018; and HR: 0.29 [0.11–0.76], *P = *.001, respectively; Table 3).

**Table 3.** Clinical and Genetic Prognostic Factors in *TERTp* Wild-Type GBM

	Univariate Analysis		Multivariate Analysis	
Prognostic factor	HR (95% CI)	*P*-value	HR (95% CI)	*P*-value
Sex (male)	2.00 (0.96–4.18)	.065	2.12 (0.98–4.61)	.058
Age (>70 years)	2.88 (1.38–6.00)	.005^*^	2.69 (1.19–6.10)	.018^*^
KPS score (≤80 points)	1.96 (0.92–4.18)	.082	0.93 (0.40–2.16)	.862
Resection (90%>)	2.24 (1.06–4.73)	.035^*^	2.05 (0.92–4.58)	.079
*PTEN* loss and/or mut	0.20 (0.08–0.46)	<.001^*^	0.29 (0.11–0.76)	.012^*^

KPS, Karnofsky Performance Status; mut, mutation.

^*^indicates statistical significance.

### *PTEN* Loss and/or Mutation is Associated With Favorable Prognoses in Patients With *TERTp*-Wild-Type GBM

**Figure 1.**
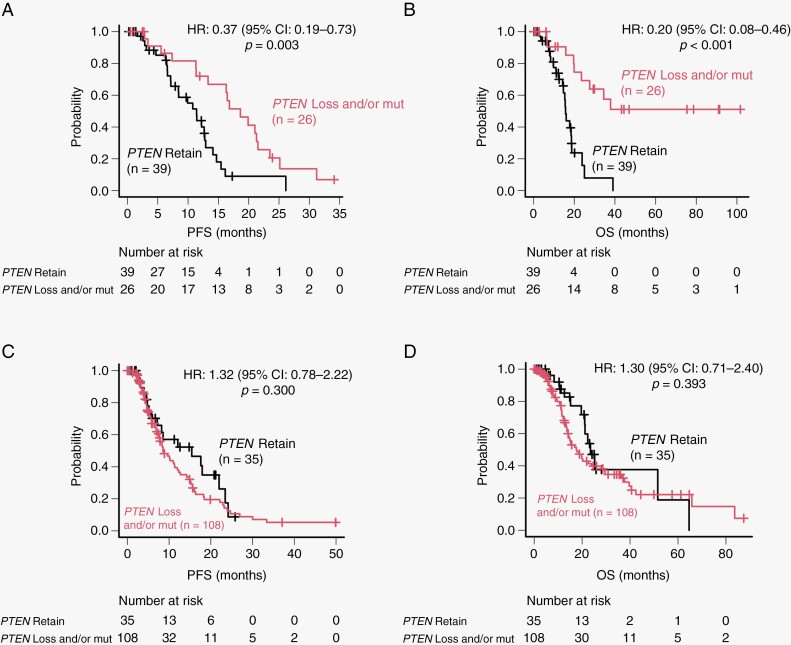
Unadjusted progression-free survival (PFS) and overall survival (OS) analyses of patients with *IDH*-wild-type glioblastoma according to *TERTp* and *PTEN* status. (A) Unadjusted PFS analysis of patients with and without *PTEN* loss and/or mutation in *IDH-* and *TERTp*-wild-type glioblastomas. (B) Unadjusted OS analysis of patients with and without *PTEN* loss and/or mutation in *IDH-* and *TERTp*-wild-type glioblastomas. (C) Unadjusted PFS analysis of patients with and without *PTEN* loss and/or mutation in *IDH-*wild-type and *TERTp*-mutant glioblastomas. (D) Unadjusted OS analysis of patients with and without *PTEN* loss and/or mutation in *IDH-*wild-type and *TERTp*-mutant glioblastomas.

**Figure 2.**
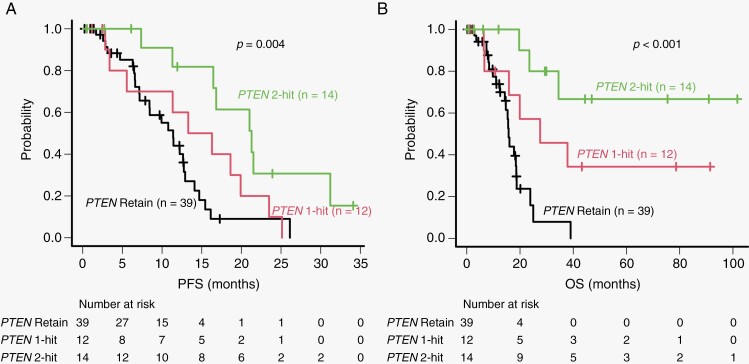
Comparison of PFS (A) and OS (B) in patients with *PTEN* 2-hit, *PTEN* 1-hit, and *PTEN* retain. Patients with *PTEN* homozygous deletion or *PTEN* mutation + loss were classified as *PTEN* 2-hit. Patients with either *PTEN* heterozygous deletion or *PTEN* mutation were classified as *PTEN* 1-hit. Patients with wild-type *PTEN* were classified as *PTEN* retain. PFS, progression-free survival; OS, overall survival.

Supplementary Table 2 compares the genetic and clinical factors of the patients with *TERTp*-wild-type GBM based on their *PTEN* status. Patients with *TERTp*-wild-type GBM with *PTEN* loss and/or mutation were younger (*P* = .011) and had higher KPS scores (*P* = .005) than those without *PTEN* loss and/or mutation. We discovered that alterations in *RB1* and *EGFR* in *TERTp*-wild-type GBMs were more common with *PTEN* loss and/or mutation than without *PTEN* loss and/or mutation (*P* = .021 and *P* = .014 for *RB1* and *EGFR*, respectively). Conversely, in *TERTp*-wild-type GBMs, *CDKN2A/B* homozygous deletion and *PDGFRA* amplification and/or mutation were more common without *PTEN* loss and/or mutation than with *PTEN* loss and/or mutation (*P* = .011 and *P* = .005, respectively). We observed a significant difference in the median PFS (18.6 and 11.4 months, respectively; *P = *.003; Figure 1A) and OS (not reached and 15.7 months, respectively; *P* < .001; Figure 1B) in patients with and without *PTEN* loss and/or mutation in *TERTp*-wild-type GBM. We classified patients with *PTEN* homozygous deletion or *PTEN* mutation + loss as *PTEN* 2-hit, those with either *PTEN* heterozygous deletion or *PTEN* mutation as *PTEN* 1-hit, and those with wild-type *PTEN* as *PTEN* retain. The PFS and OS were significantly longer for *PTEN* 2-hit than for *PTEN* 1-hit and *PTEN* retain (*P* = .004 and *P* < .001 for PFS and OS, respectively; Figure 2A, 2B). However, the median PFS (8.5 and 15.4 months, respectively; *P = *.300; Figure 1C) and OS (17.8 and 23.5 months, respectively; *P = *.393; Figure 1D) were not significantly different when we compared *TERTp*-mutant GBMs with and without *PTEN* loss and/or mutation. These results indicate that *PTEN* loss and/or mutation is a good prognostic factor that depends on *TERTp* status.

### Unsupervised Hierarchical Cluster Analysis of *TERTp*-*W*ild-Type GBM

**Figure 3.**
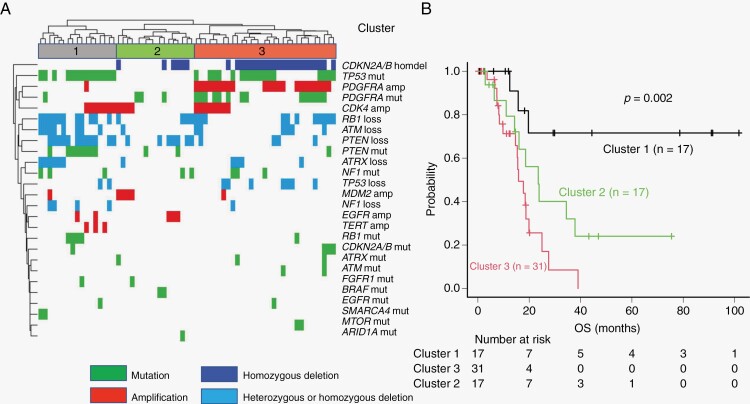
Risk stratification of patients with *IDH*- and *TERTp*-wild-type glioblastomas based on unsupervised hierarchical clustering analysis. (A) Results of unsupervised hierarchical clustering analysis in 65 *IDH*- and *TERTp*-wild-type glioblastomas. (B) Unadjusted Kaplan–Meier overall survival (OS) curves for each cluster.

We performed an unsupervised hierarchical cluster analysis on the 65 *TERTp*-wild-type GBMs, which revealed 3 major distinct groups. One major cluster (cluster 1) was characterized by alterations in *PTEN* and *TP53* and lack of *CDKN2A/B* homozygous deletion and *PDGFRA* alterations (Figure 3A). The second major cluster (cluster 2) was characterized by lack of alterations in *PDGFRA* and *TP53* (Figure 3A). The third major cluster (cluster 3) was characterized by alterations in *PDGFRA* and *TP53*, homozygous deletion of *CDKN2A/B*, and lack of alterations in *PTEN* (Figure 3A). Additionally, we compared the clinical features among clusters 1, 2, and 3. The average age in cluster 1 was 57.18 years, and these patients were significantly younger than those in clusters 2 and 3 (*P* = .002) (Supplementary Table 3). However, we did not detect any differences in clinical factors, including sex, KPS score, EOR, and chemoradiotherapy, among the clusters (Supplementary Table 3). However, the OS was significantly longer for cluster 1 than for clusters 2 and 3 (*P* = .002; Figure 3B).

## Discussion

GBM is the most frequent and deadly primary brain tumor; it is heterogeneous with a wide mutational spectrum.^2–4^ In an effort to better classify *IDH*- and *TERTp*-wild-type GBMs, we used a custom gene panel to genotype these neoplasms. Herein, we demonstrated the impact of *PTEN* loss and/or mutation as a favorable prognostic marker of *IDH*- and *TERTp*-wild-type GBM. Additionally, we revealed the molecular genetic profile in Japanese patients with *IDH*- and *TERTp*-wild-type GBM and 3 major distinct groups of *IDH*- and *TERTp*-wild-type GBMs.

In the current study, no distinct difference in survival was observed for patients with *TERTp*-wild-type GBMs or *TERTp*-mutant GBMs. Similar findings of a lack of prognostic significance of *TERTp* mutations among *IDH*-wild-type GBMs were previously reported.^21,26^ Another report showed that *PDGFRA* amplification and *TP53* loss were more common in *TERTp*-wild-type GBMs than in *TERTp*-mutant GBMs, while *EGFR* amplification and *PTEN* loss were more commonly observed in *TERTp*-mutant GBMs than in *TERTp*-wild-type GBMs.^19^ These findings are consistent with our results. Recently, Williams et al. reported that *TERTp*-wild-type GBMs showed frequent mutations in the *PI3K* pathway and *BAF* complex gene family (*ATRX*, *SMARCA4*, *SMARCB1*, and *ARID1A*),^22^ while our results showed no differences in the frequencies of *ATRX*, *SMARCA4*, and *ARID1A* mutations between *TERTp*-wild-type and *TERTp*-mutant GBMs. Unfortunately, our study did not include an analysis of mutations in the *PI3K* pathway. Diplas et al. identified novel molecular subgroups of *TERTp*-wild-type GBMs, including a telomerase-positive subgroup driven by *TERT* structural rearrangements, and an alternative lengthening of telomeres-positive subgroup with mutations in *ATRX* or *SMARCAL1*.^21^ Collectively, these data suggest that *TERTp*-wild-type GBMs are genetically distinct from *TERTp*-mutant GBMs.

Moreover, the frequencies of *TERTp*-wild-type GBM were higher in the Japanese group than in groups from other countries. Thus, we hypothesized that *IDH*- and *TERTp*-wild-type GBMs in our cohort have distinct molecular profiles and clinical characteristics. Herein, the frequencies of altered genes within *IDH*- and *TERTp*-*wild*-type GBMs were *TP53*: 57%, *PDGFRA*: 45%, *CDKN2A/B*: 43%, *RB1*: 43%, *PTEN*: 40%, and *EGFR*:11%. Recently, 2 US reports showed that the frequencies of altered genes in *IDH*- and *TERTp*- wild-type GBMs were *TP53*: 24%–69%, *PDGFRA*: 8%, *CDKN2A/B*: 12%–19%, *RB1*: 4%, *PTEN*: 13%–16%, and *EGFR*: 28%–31%,^21,22^ which were different from the frequencies observed in our study. These discrepancies may be caused by racial differences. Interestingly, in our study, when we excluded cluster 3, which was characterized by alterations in *PDGFRA* and *TP53*, the homozygous deletion of *CDKN2A/B*, and lack of *PTEN* alterations, the frequencies of altered *PDGFRA* and *CDKN2A/B* genes were similar to those of previous reports.^21,22^ Therefore, cluster 3 might include a specific subgroup of Japanese patients. For *IDH*- and *TERTp*-wild-type GBMs, *CDKN2A/B* homozygous deletion and *PDGFRA* amplification and/or mutation were associated with worse OS, while *PTEN* loss and/or mutation was a significant predictor of favorable outcomes. Only *PTEN* loss and/or mutation was an independent prognostic indicator in the multivariate analyses. Surprisingly, when examined according to Knudson’s “two-hit” hypothesis,^27^ patients with *PTEN* 2-hit such as *PTEN* homozygous deletion or *PTEN* mutation + loss had a better prognosis than patients with *PTEN* 1-hit and *PTEN* retain. Liu et al. showed that *CDKN2A/B* homozygous deletion is a poor prognostic marker for *IDH*- and *TERTp*-wild-type GBMs, which is consistent with our findings.^23^ To date, the prognostic value of *PTEN* alterations in GBM remains controversial. While several studies have reported the poor prognostic impact of *PTEN* alterations in GBM,^28,29^ other studies have reported favorable prognostic impact.^30–33^*PTEN* is a lipid phosphatase with a canonical role in dampening the PI3K/Akt-1 signaling pathway; hence, loss of *PTEN* driven by genetic alterations or epigenetic silencing has oncogenic consequences during gliomagenesis.^34^ In contrast, recent reports have shown that *PTEN* loss can be associated with a more favorable prognosis, since it leads to a better response to chemotherapy or radiotherapy.^35,36^ Moreover, in this study, patients with *TERTp*-wild-type GBM with *PTEN* loss and/or mutation were younger and had higher KPS scores than those without *PTEN* loss and/or mutation. Previous reports indicated that young age and high KPS scores are favorable prognostic factors.^37,38^ These findings would explain why *PTEN* alterations predict favorable outcomes in GBM. Interestingly, for *TERTp*-mutant GBMs, *PTEN* loss and/or mutation was not a predictor of OS in our study. Because *TERTp*-wild-type GBMs are genetically distinct from *TERTp*-mutant GBMs, the prognostic impact of *PTEN* may depend on *TERTp* status. To the best of our knowledge, the prognostic impact of *PTEN* loss and/or mutation in *IDH*- and *TERTp*-wild-type GBMs has not been documented.

Using hierarchical molecular classification of *IDH*- and *TERTp*-wild-type GBMs, we revealed 3 distinct groups. One major cluster (cluster 1) was characterized by loss and/or mutation in *PTEN* and *TP53*, and lack of *CDKN2A/B* homozygous deletion and *PDGFRA* amplification and/or mutation. Interestingly, cluster 1 was significantly associated with younger age and favorable prognosis. Previous reports showed that *TP53* mutations can be associated with favorable prognosis.^39–41^ Thus, our finding that cluster 1, with *PTEN* loss and/or mutation and *TP53* mutations, had a favorable prognosis is reasonable. Interestingly, evidence points toward an interplay between *PTEN* and *TP53* in which they regulate each other at the transcriptional and protein levels.^42,43^ Our most striking finding was that *PTEN*, *TP53*, *CDKN2A/B*, and *PDGFRA* are important driver genes in the molecular classification of *IDH*- and *TERTp*-wild-type GBM. Furthermore, the combination of these 4 genes predicts individual outcomes in patients with *IDH*- and *TERTp*-wild-type GBM.

This study had some limitations. First, this was a retrospective study susceptible to selection biases. Second, epigenetic silencing of the *PTEN* promoter has been identified as an alternative method for gene inactivation.^43^ However, our study did not include an epigenetic analysis of *PTEN*.

## Conclusions

We report that *TERTp*-wild-type GBMs are genetically distinct from *TERTp*-mutant GBMs, and *PTEN* loss and/or mutation is a good prognostic indicator in *IDH*- and *TERTp*-wild-type GBM. We recommend the incorporation of 4 combined genes (*PTEN*, *TP53*, *CDKN2A/B*, and *PDGFRA*) in the molecular stratification of *IDH*- and *TERTp*-wild-type GBMs. Such stratification will likely provide precise information to patients and help influence bedside decisions.

## Supplementary Material

vdad078_suppl_Supplementary_Materials

## Data Availability

All data used and analyzed in the current study are available from the corresponding author upon reasonable request.
