# Supplementary material for: Favorable prognostic impact of phosphatase and tensin homolog alterations in wild-type isocitrate dehydrogenase and telomerase reverse transcriptase promoter glioblastoma
Source: Neurooncol Adv. 2023 Jun 28;5(1):vdad078. doi: 10.1093/noajnl/vdad078 (PMC10390081; doi:10.1093/noajnl/vdad078)
Supplement: vdad078_suppl_Supplementary_Materials [file vdad078_suppl_supplementary_materials.zip › KRT64745e8f75be2.docx]

## KEY RESOURCES TABLE

| REAGENT or RESOURCE | SOURCE | IDENTIFIER |
| --- | --- | --- |
| Critical Commercial Assays | | |
| Maxwell 16 FFPE Tissue LEV DNA Purification Kit | Promega, Madison, WI, United States | AS1130 |
| Qubit dsDNA BR Assay Kit | Life Technologies, Grand Island, NY, United States | Q32850 |
| QIAseq DNA QuantiMIZE Kit | QIAGEN, Reston, VA, United States | Cat. No. / ID:  333414 |
| QIAseq Targeted DNA Custom Panel | QIAGEN, Reston, VA, United States | Cat. No. / ID: 333525 |
| Software and Algorithms | | |
| EZR | Saitama Medical Center, Jichi Medical University, Saitama, Japan | https://cran.r-project.org |
| a graphical user interface of R software | The R Foundation for Statistical Computing, Vienna, Austria | https://www.r-project.org/foundation/ |
| QIAGEN Web Portal service | QIAGEN, Reston, VA, United States | https://www.qiagen.com/ |
