# Supplementary material for: Favorable prognostic impact of phosphatase and tensin homolog alterations in wild-type isocitrate dehydrogenase and telomerase reverse transcriptase promoter glioblastoma
Source: Neurooncol Adv. 2023 Jun 28;5(1):vdad078. doi: 10.1093/noajnl/vdad078 (PMC10390081; doi:10.1093/noajnl/vdad078)
Supplement: vdad078_suppl_Supplementary_Materials [file vdad078_suppl_supplementary_materials.zip › Supplementary fig. 1.pptx]

## Slide 1
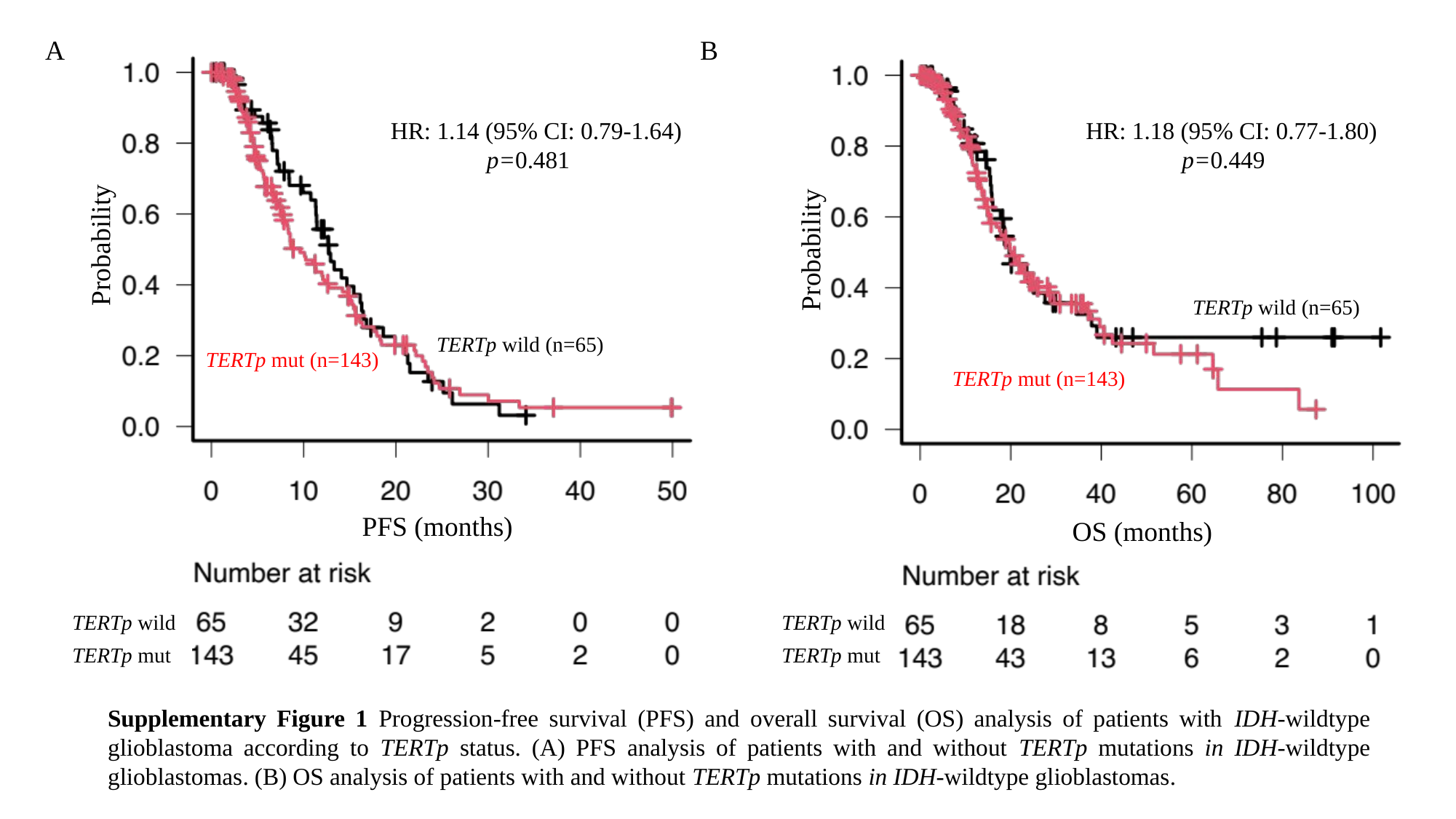

A
B
HR: 1.18 (95% CI: 0.77-1.80)
 p=0.449
HR: 1.14 (95% CI: 0.79-1.64)
 p=0.481
Probability
Probability
TERTp wild (n=65)
TERTp wild (n=65)
TERTp mut (n=143)
TERTp mut (n=143)
PFS (months)
OS (months)
TERTp wild
TERTp wild
TERTp mut
TERTp mut
Supplementary Figure 1 Progression-free survival (PFS) and overall survival (OS) analysis of patients with IDH-wildtype glioblastoma according to TERTp status. (A) PFS analysis of patients with and without TERTp mutations in IDH-wildtype glioblastomas. (B) OS analysis of patients with and without TERTp mutations in IDH-wildtype glioblastomas.
