# Supplementary material for: Favorable prognostic impact of phosphatase and tensin homolog alterations in wild-type isocitrate dehydrogenase and telomerase reverse transcriptase promoter glioblastoma
Source: Neurooncol Adv. 2023 Jun 28;5(1):vdad078. doi: 10.1093/noajnl/vdad078 (PMC10390081; doi:10.1093/noajnl/vdad078)
Supplement: vdad078_suppl_Supplementary_Materials [file vdad078_suppl_supplementary_materials.zip › Supplementary Fig. 2.pptx]

## Slide 1
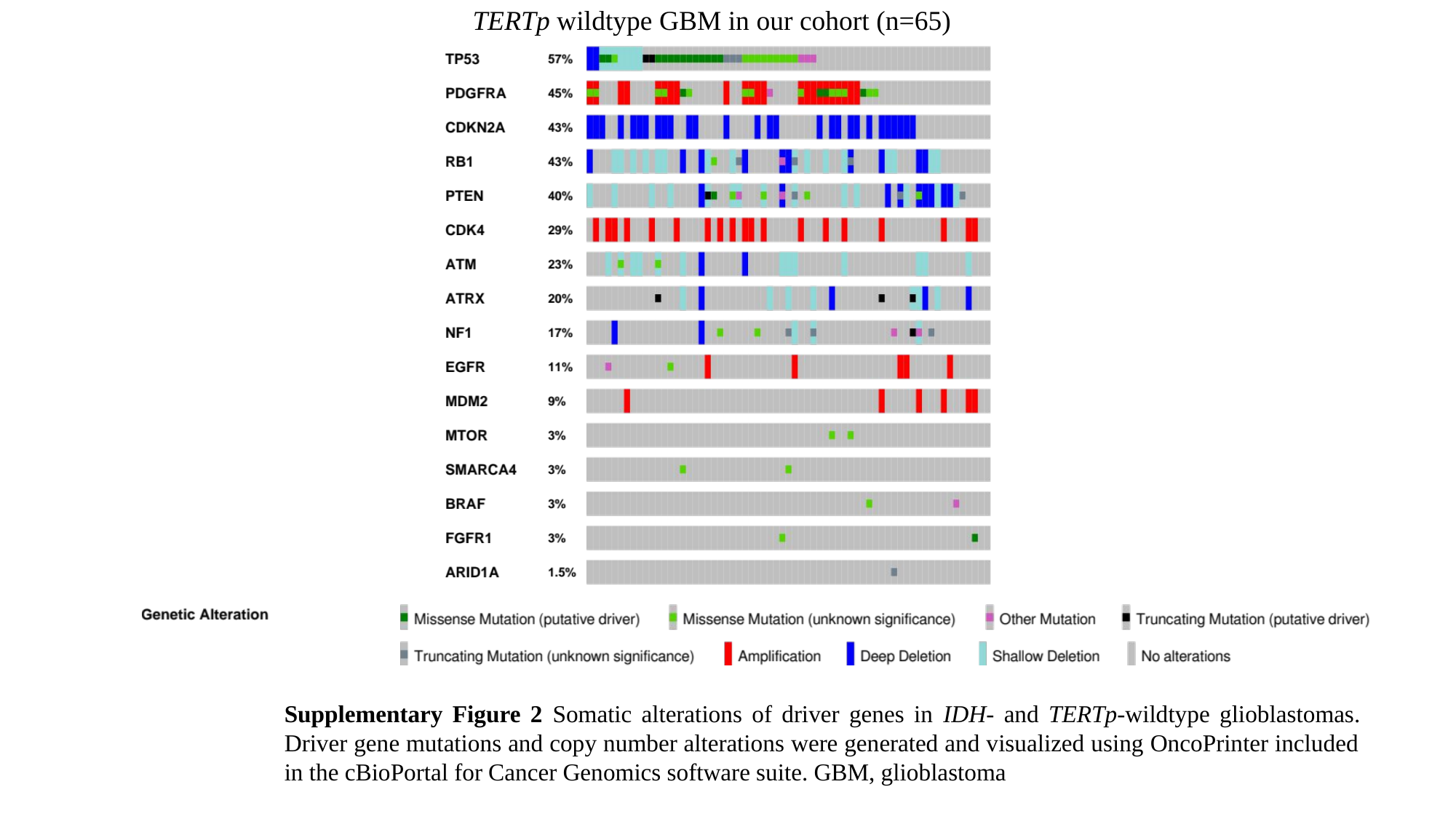

TERTp wildtype GBM in our cohort (n=65)
Supplementary Figure 2 Somatic alterations of driver genes in IDH- and TERTp-wildtype glioblastomas. Driver gene mutations and copy number alterations were generated and visualized using OncoPrinter included in the cBioPortal for Cancer Genomics software suite. GBM, glioblastoma
