# Supplementary material for: Favorable prognostic impact of phosphatase and tensin homolog alterations in wild-type isocitrate dehydrogenase and telomerase reverse transcriptase promoter glioblastoma
Source: Neurooncol Adv. 2023 Jun 28;5(1):vdad078. doi: 10.1093/noajnl/vdad078 (PMC10390081; doi:10.1093/noajnl/vdad078)
Supplement: vdad078_suppl_Supplementary_Materials [file vdad078_suppl_supplementary_materials.zip › Supplementary Table 2.docx]

Supplementary Table 2. Background of patients with and without *PTEN* alterations in *TERTp*-wildtype GBMs.

| Prognostic factor | | All (n=65) | *PTEN* wild  (n=39) | *PTEN* alteration  (n=26) | *p*-value |
| --- | --- | --- | --- | --- | --- |
| Sex | male | 36 (55.4%) | 24 (61.5%) | 12 (46.2%) | 0.309 |
|  | female | 29 (44.6%) | 15 (38.5%) | 14 (53.8%) |  |
| Age | 70 years> | 34 (52.3%) | 15 (38.5%) | 19 (73.1%) | 0.011* |
|  | 70 years≤ | 31 (47.7%) | 24 (61.5%) | 7 (26.9%) |  |
| KPS score | 80 points≤ | 28 (43.1%) | 11 (28.2%) | 17 (65.4%) | 0.005* |
|  | 80 points> | 37 (56.9%) | 28 (71.8%) | 9 (34.6%) |  |
| Resection | 90 %≤ | 27 (41.5%) | 13 (33.3%) | 14 (53.8%) | 0.127 |
|  | 90 %> | 38 (58.5%) | 26 (66.7%) | 12 (46.2%) |  |
| Chemoradiotherapy | Yes | 60 (92.3%) | 34 (87.2%) | 26 (100%) | 0.078 |
|  | No | 5 (7.7%) | 5 (12.8%) | 0 (0%) |  |
| *CDKN2A/B* homdel | | 28 (43.1%) | 22 (56.4%) | 6 (23.1%) | 0.011* |
| *NF1* loss and/or mut | | 11 (16.9%) | 6 (15.4%) | 5 (19.2%) | 0.743 |
| *RB1* loss and/or mut | | 28 (43.1%) | 12 (30.8%) | 16 (61.5%) | 0.021* |
| *PDGFRA* amp and/or mut | | 29 (44.6%) | 23 (59.0%) | 6 (23.1%) | 0.005* |
| *TP53* loss and/or mut | | 37 (56.9%) | 24 (61.5%) | 13 (50.0%) | 0.446 |
| *EGFR* amp and/or mut | | 7 (10.8%) | 1 (2.6%) | 6 (23.1%) | 0.014* |
| *ATRX* loss and/or mut | | 13 (20.0%) | 9 (23.1%) | 4 (15.4%) | 0.538 |

KPS, Karnofsky Performance Status; mut, mutation; amp, amplification; homdel, homozygous deletion

*indicates statistical significance.
