# Supplementary material for: Favorable prognostic impact of phosphatase and tensin homolog alterations in wild-type isocitrate dehydrogenase and telomerase reverse transcriptase promoter glioblastoma
Source: Neurooncol Adv. 2023 Jun 28;5(1):vdad078. doi: 10.1093/noajnl/vdad078 (PMC10390081; doi:10.1093/noajnl/vdad078)
Supplement: vdad078_suppl_Supplementary_Materials [file vdad078_suppl_supplementary_materials.zip › Supplementary Table 3.docx]

Supplementary Table 3. Clinical features of *TERTp* wildtype glioblastomas according to subtypes by unsupervised hierarchical cluster analysis

|  | | Cluster 1 (n=17) | Cluster 2 (n=17) | Cluster 3 (n=31) | *p* value |
| --- | --- | --- | --- | --- | --- |
| Age | | 57.18±17.95 | 63.65±15.87 | 73.90±11.44 | <0.001* |
| Sex | male | 7 (41.2%) | 12 (70.6%) | 17 (54.8%) | 0.246 |
|  | female | 10 (58.8%) | 5 (29.4%) | 14 (45.2%) |  |
| KPS score | 80 points≤ | 11 (64.7%) | 6 (35.3%) | 11 (35.5%) | 0.141 |
|  | 80 points> | 6 (35.3%) | 11 (64.7%) | 20 (64.5%) |  |
| Resection | 90 %≤ | 8 (47.1%) | 9 (52.9%) | 10 (35.5%) | 0.352 |
|  | 90 %> | 9 (52.9%) | 8 (47.1%) | 21 (64.5%) |  |
| Chemoradiotherapy | Yes | 16 (94.1%) | 16 (94.1%) | 28 (90.3%) | 1.000 |
|  | No | 1 (5.9%) | 1 (5.9%) | 3 (9.7%) |  |
| Representative genetic features | *CDKN2A/B* | retain | homdel | homdel |  |
|  | *PDGFRA* | retain | retain | mut / amp |  |
|  | *PTEN* | mut / loss | mut / loss | retain |  |
|  | *TP53* | mut / loss | retain | mut / loss |  |

KPS, Karnofsky Performance Status; mut, mutation; amp, amplification; homdel, homozygous deletion

*indicates statistical significance.
