# Supplementary material for: Favorable prognostic impact of phosphatase and tensin homolog alterations in wild-type isocitrate dehydrogenase and telomerase reverse transcriptase promoter glioblastoma
Source: Neurooncol Adv. 2023 Jun 28;5(1):vdad078. doi: 10.1093/noajnl/vdad078 (PMC10390081; doi:10.1093/noajnl/vdad078)
Supplement: vdad078_suppl_Supplementary_Materials [file vdad078_suppl_supplementary_materials.zip › Supplementary Table. 1.docx]

Supplementary Table 1. Genetic prognostic factors in *TERTp* mutant GBM.

|  | Univariate analysis | | Multivariate analysis | |
| --- | --- | --- | --- | --- |
| Genetic marker | HR (95% CI) | *p*-value | HR (95% CI) | *p*-value |
| *CDKN2A/B* homdel | 0.93 (0.58-1.50) | 0.779 | 0.85 (0.50-1.43) | 0.533 |
| *NF1* loss and/or mut | 1.08 (0.64-1.84) | 0.769 | 1.20 (0.68-2.10) | 0.526 |
| *RB1* loss and/or mut | 0.97 (0.59-1.58) | 0.895 | 1.02 (0.61-1.69) | 0.943 |
| *EGFR* amp and/or mut | 0.89 (0.55-1.43) | 0.616 | 1.02 (0.61-1.69) | 0.946 |
| *PDGFRA* amp and/or mut | 2.11 (1.03-4.31) | 0.041* | 2.26 (1.04-4.91) | 0.039* |
| *TP53* loss and/or mut | 1.04 (0.63-1.70) | 0.884 | 1.06 (0.62-1.80) | 0.834 |
| *PTEN* loss and/or mut | 1.30 (0.71-2.40) | 0.394 | 1.29 (0.67-2.48) | 0.454 |

mut, mutation; homdel, homozygous deletion; amp, amplification

*indicates statistical significance.
